# The Aertex Cellular Clothing Factory, Swindon

**Published:** 1906-09-08

**Authors:** 


### THE AERTEX CELLULAR CLOTHING FACTORY, SWINDON.

It is scarcely necessary to remark that the object of clothes is to prevent the radiation of heat from our bodies, and they accomplish this end largely by surrounding the body with layers of air. Cold air blowing over the surface of the skin lowers the temperature of the skin because the air as it passes receives some of the warmth of the body, but air retained in contact with the body by clothes soon becomes warmed, and the air being a bad conductor of heat, prevents radiation into the colder air outside the clothes. The value of air as a retainer of warmth is seen by the appearance of birds on a cold day. They puff out their feathers and thus surround themselves with a covering apparently two or three times the thickness of that they seem to possess on other occasions. The increase in thickness, it is obvious, is due to greater space, and consequently more air, between the feathers. A desirable clothing provides warmth without undue weight. The warmth, yet lightness of a feathery covering, is well illustrated by an eider-down quilt. Cellular clothing is designed much upon the principle of feathers, that is to say, the aim of its inventors has been to provide for as much air by means of as little texture as possible in the garments, and thus increase the power for warmth with a diminution of weight. Cellular clothing, no doubt, has one other advantage. It must allow the escape of perspiration more readily than closely woven clothing.

The factory is pleasantly situated on the outskirts of Swindon, green fields stretching away from it towards the Wiltshire Downs. The day was one of the hottest at the time of our visit, yet in spite of the number of hands present the air was refreshingly cool. This is due to the presence of the plenum system of ventilation, which propels fresh air to every part of the building. In winter the air is warmed. Until this system was adopted it was found impossible to maintain an equable temperature throughout the work-rooms in cold weather. Not only the efficiency of the ventilation, but the cleanliness, tidiness, and contented appearance of the workers were striking features.

